# Weighted norm inequalities for Toeplitz type operators associated to generalized Calderón–Zygmund operators

**DOI:** 10.1186/s40064-016-3022-7

**Published:** 2016-08-17

**Authors:** Yongli Tang, Tao Ban

**Affiliations:** 1 College of Computer Science and Technology, Henan Polytechnic University, Jiaozuo, 454003 China; 2 College of Mathematics and Informatics, Henan Polytechnic University, Jiaozuo, 454003 China

**Keywords:** Weighted norm inequality, Toeplitz type operator, Generalized Calderón–Zygmund operator, Weighted Lipschitz function, Fractional integral, 42B25, 42B20

## Abstract

Let $T_1$ be a generalized Calderón–Zygmund operator or $\pm I$ ( the identity operator), let $T_2$ and $T_3$ be the linear operators, and let $T_3=\pm I$. Denote the Toeplitz type operator by

$$\begin{aligned} T^b=T_1M^bI_\alpha T_2+T_3I_\alpha M^b T_4, \end{aligned}$$

where $M^bf=bf,$ and $I_\alpha$ is fractional integral operator. In this paper, we establish the sharp maximal function estimates for $T^b$ when *b* belongs to weighted Lipschitz function space, and the weighted norm inequalities of $T^b$ on weighted Lebesgue space are obtained.

## Introduction and results

As the development of the singular integral operators, their commutators have been well studied (Coifman et al. 1976; Harboure et al. 1997; Lin et al. 2015). Coifman et al. (1976) proved that the commutators [*b*, *T*], which generated by Calderón–Zygmund singular integral operators and BMO functions, are bounded on $L^p({\mathbb {R}}^n)$ for $1<p<\infty .$ Chanillo (1982) obtained a similar result when Calderón–Zygmund singular integral operators are replaced by the fractional integral operators. Recently, some Toeplitz type operators associated to the singular integral operators are introduced, and the boundedness for the operators generated by singular integral operators and BMO functions and Lipschitz functions are obtained (see Lin and Lu 2006; Lu and Mo 2009).

The following generalized Calderón–Zygmund operator was introduced by Chang et al. (2007).

### **Definition 1**

Let ${\mathcal {S}}({\mathbb {R}}^n)$ be the space of all Schwartz functions on ${\mathbb {R}}^n$ and ${\mathcal {S'}}({\mathbb {R}}^n)$ its dual space, the class of all tempered distributions on ${\mathbb {R}}^n.$ Suppose that $T:{\mathcal {S}}({\mathbb {R}}^n)\rightarrow {\mathcal {S}}'({\mathbb {R}}^n)$ is a linear operator with kernel $K(\cdot ,\cdot )$ defined initially by

$$\begin{aligned} T(f)(x)=\int _{{\mathbb {R}}^n}K(x,y)f(y)dy,\quad f\in C_c^\infty ({\mathbb {R}}^n),\quad x\notin {\text{ supp }}f. \end{aligned}$$

The operator *T* is called a generalized Calderón–Zygmund operator provided the following three conditions are satisfied:

1.  *T* can be extended as a continuous operator on $L^2({\mathbb {R}}^n)$;
2.  *K* is smooth away from the diagonal $\{(x,y):x=y\}$ with
  $$\begin{aligned} \int _{|x-y|>2|z-y|}\Big (|K(x,y)-K(x,z)|+|K(y,x)-K(z,x)|\Big )dx\le C, \end{aligned}$$
  where $C>0$ is a constant independent of *y* and *z*;
3.   There is a sequence of positive constant numbers $\{C_j\}$ such that for each $j\in {N},$
  $$\begin{aligned} \bigg (\int _{2^j|z-y|\le |x-y|<2^{j+1}|z-y|}|K(x,y)-K(x,z)|^\gamma dx\bigg )^{1/\gamma }\le C_j(2^j|z-y|)^{-n/\gamma '} \end{aligned}$$
  and
  $$\begin{aligned} \bigg (\int _{2^j|z-y|\le |x-y|<2^{j+1}|z-y|}|K(y,x)-K(z,x)|^\gamma dx\bigg )^{1/\gamma }\le C_j(2^j|z-y|)^{-n/\gamma '} \end{aligned}$$
  where $(\gamma ,\gamma ')$ is a fixed pair of positive numbers with $1/\gamma +1/\gamma '=1$ and $1<\gamma '<2.$

If we compare the generalized Calderón–Zygmund operator with the classical Calderón–Zygmund operator, whose kernel *K*(*x*, *y*) enjoys the conditions

$$\begin{aligned} |K(x,y)|\le C|x-y|^{-n} \end{aligned}$$

and

$$\begin{aligned} |K(x,y)-K(x,z)|+ |K(y,x)-K(z,x)|\le C|x-y|^{-n}\bigg (\frac{|z-y|}{|x-y|}\bigg )^\delta , \end{aligned}$$

where $|x-y|>2|z-y|$ for some $\delta >0,$ we can find out that the classical Calderón–Zygmund operator is a generalized Calderón–Zygmund operator defined above with $C_j=2^{-j\delta },j\in N,$ and any $1<\gamma <\infty .$

Let *b* be a locally integrable function on ${\mathbb {R}}^n.$ The Toeplitz type operator associated to generalized Calderón–Zygmund operator and fractional integral operator $I_\alpha$ is defined by

$$\begin{aligned} T^b=T_1M^bI_\alpha T_2+T_3I_\alpha M^b T_4, \end{aligned}$$

where $T_1$ is the generalized Calderón–Zygmund operator or $\pm I$ (the identity operator), $T_2$ and $T_4$ are the linear operators, $T_3=\pm I,$ and $M^b f=bf.$

Note that the commutators $[b,I_\alpha ](f)= bI_\alpha (f)-I_\alpha (bf)$ are the particular operators of the Toeplitz type operators $T^b.$ The Toeplitz type operators $T^b$ are the non-trivial generalizations of these commutators.

It is well known that the commutators of fractional integral have been widely studied by many authors. Paluszyński (1995) showed that $b\in Lip_\beta ({\mathbb {R}}^n)(0<\beta <1)$ (homogeneous Lipschitz space) if and only if $[b,I_\alpha ]$ is bounded from $L^p({\mathbb {R}}^n)$ to $L^q({\mathbb {R}}^n),$ where $1<p<{n}/({\alpha +\beta })$ and ${1}/{q}={1}/{p}-({\alpha +\beta })/{n}.$ When *b* belongs to the weighted Lipschitz spaces $Lip_{\beta }(\omega ),$ Hu and Gu (2008) proved that $[b,I_\alpha ]$ is bounded from $L^p(\omega )$ to $L^q(\omega ^{1-(1-\alpha /n)q})$ for $1/q=1/p-(\alpha +\beta )/n$ with $1<p<n/(\alpha +\beta ).$ A similar result obtained when $I_\alpha$ is replaced by the generalized fractional integral operator (Hu et al. 2013).

This paper investigates the boundedness of the Toeplitz type operator associated to generalized Calderón–Zygmund operator, fractional integral operator $I_\alpha$ and weighted Lipschitz function on weighted Lebesgue space. The main result is as follows.

### **Theorem 1**

*Suppose that*$$T^b$$*is a Toeplitz type operator associated to generalized Calderón–Zygmund operator and fractional integral operator*$$I_\alpha $$, *and*$$b\in Lip_{\beta }(\omega )(0<\beta <1).$$*Let*$$0<\alpha<n,\gamma '<p<n/(\alpha +\beta ),1/q=1/p-(\alpha +\beta )/n,\{jC_j\}\in l^1,\omega ^{q/p}\in A_1$$*and the critical index of*$$\omega $$*for the reverse Hölder condition*$$r_\omega >\frac{(q-1)\gamma '}{q-\gamma '}.$$*If*$$T^1(f)=0$$*for any*$$f\in L^{p}(\omega ),$$$$T_2$$*and*$$T_4$$*are the bounded operators on*$$L^{p}(\omega ),$$*then there exists a constant*$$C>0$$*such that,*

$$\begin{aligned} \Vert T^b(f)\Vert _{L^{q}(\omega ^{1-(1-\alpha /n)q})}\le C\Vert b\Vert _{Lip_{\beta }(\omega )}\Vert f\Vert _{L^p(\omega )}. \end{aligned}$$

Noticing that the classical Calderón–Zygmund operator is a generalized Calderón–Zygmund operator with $C_j=2^{-j\delta }(j\in {\mathbb {N}})$ and any $1<\gamma <\infty .$ Then we can obtain the following result as a corollary.

### **Corollary 1**

*LetTbe a classical Calderón–Zygmund operator, *$0<\beta<1,1<p<n/(\alpha +\beta ),1/q=1/p-(\alpha +\beta )/n,$*and*$\omega ^{q/p}\in A_1.$*If*$b\in Lip_\beta (\omega ),$*then*$T^b$*is bounded from*$L^p(\omega )$*to*$L^{q}(\omega ^{1-(1-\alpha /n)q}).$

The paper is organized as follows. Section  introduces some notation and definitions, and recalls some preliminary results. Section  establishes the sharp estimates for Toeplitz type operators. Section  gives the proof of Theorem 1.

In this paper, we shall use the symbol $$A\lesssim B$$ to indicate that there exists a universal positive constant *C*,  independent of all important parameters, such that $$A\le CB.$$$$A\thickapprox B$$ means that $$A\lesssim B$$ and $$B\lesssim A.$$

## Some preliminaries

A weight $$\omega $$ is a nonnegative, locally integrable function on $${\mathbb {R}}^n.$$ Let $$ B = B_r(x_0)$$ denote the ball with the center $$x_0$$ and radius *r*, and let $$\lambda B=B_{\lambda r}(x_0)$$ for any $$\lambda >0.$$ For a given weight function $$\omega $$ and a measurable set *E*, we also denote the Lebesgue measure of *E* by |*E*| and set weighted measure $$\omega (E)=\int _E\omega (x) dx.$$ For any given weight function $$\omega $$ on $${\mathbb {R}}^n,$$$$0<p<\infty ,$$ denote by $$L^p(\omega )$$ the space of all function *f* satisfying

$$\begin{aligned} \Vert f\Vert _{L^p(\omega )}=\left( \int _{{\mathbb {R}}^n}|f(x)|^p\omega (x)dx\right) ^{1/p}<\infty . \end{aligned}$$

### **Definition 2**

(Muckenhoupt 1972) Let $1<p<\infty .$ We say $\omega \in A_p$ if

$$\begin{aligned} \sup _{B}\left( \frac{1}{|B|}\int _{B}\omega (x)dx\right) \left( \frac{1}{|B|}\int _{B}\omega (x)^{-\frac{1}{p-1}}dx\right) ^{p-1}<\infty , \end{aligned}$$

where the supremum is considered over all ball $B\subset {\mathbb {R}}^n$ and, $\omega \in A_1$ if

$$\begin{aligned} M\omega (x)\thickapprox \omega (x)\ \ {\text{ a.e. }}\ x\in {\mathbb {R}}^n . \end{aligned}$$

### **Definition 3**

(Muckenhoupt and Wheeden 1974) A weight function $$\omega $$ belongs to $$ A_{p,q}$$ for $$1<p< q<\infty ,$$ if for every ball *B* in $${\mathbb {R}}^n,$$ such that

$$\begin{aligned} \left( \frac{1}{|B|}\int _B\omega (y)^{-p'}dy\right) ^{1/p'}\left( \frac{1}{|B|}\int _B\omega (y)^qdy\right) ^{1/q}<\infty , \end{aligned}$$

where $p'$ denotes the conjugate exponent of $p>1;$ that is, $1/p+1/p'=1.$

From the definition of $$ A_{p,q},$$ we can get that

1 $$\begin{aligned} \omega \in A_{p,q},\ {\text{ iff }} \ \omega ^q\in A_{1+q/p'}. \end{aligned}$$

### **Definition 4**

(García-Cuerva and Rubio de Francia 1985) A weight function $\omega$ belongs to the reverse Hölder class $RH_s$ if there exists constant $s>1$ such that the following reverse Hölder inequality

$$\begin{aligned} \left( \frac{1}{|B|}\int _B\omega (x)^sdx\right) ^{1/s}\lesssim \frac{1}{|B|}\int _B\omega (x)dx \end{aligned}$$

holds for every ball $B\subset {\mathbb {R}}^n.$

It is well known that if $\omega \in A_p$ with $1< p<\infty ,$ then $\omega \in A_r$ for all $r>p,$ and $\omega \in A_q$ for some $1<q<p.$ If $\omega \in A_p$ with $1\le p<\infty ,$ then there exists $r>1$ such that $\omega \in RH_r.$ It follows directly from Hölder’s inequality that $\omega \in RH_r$ implies $\omega \in RH_s$ for all $1<s<r.$ Moreover, if $\omega \in RH_r,r>1,$ then we have $\omega \in RH_{r+\epsilon }$ for some $\varepsilon >0.$ We write $r_\omega =\sup \{r>1: \omega \in RH_r\}$ to denote the critical index of $\omega$ for the reverse Hölder condition.

### **Lemma 1**

(García-Cuerva and Rubio de Francia 1985) *The following results about weight function are right.*

(i) *Suppose*$$\omega \in A_1.$$*Then*
  2 $$\begin{aligned} |B|\underset{x\in B}{{\text{ inf }}} \omega (x) \thickapprox \omega (B). \end{aligned}$$
(ii) *Let*$1\le p<\infty ,$*and*$\omega \in A_p.$*Then, for any ballBand any*$\lambda >1,$
  3 $$\begin{aligned} \omega (\lambda B)\lesssim \lambda ^{pn}\omega (B). \end{aligned}$$

Next, we shall recall the definition of the Hardy-Littlewood maximal operator and several variants, the fractional integral operator and some function spaces.

### **Definition 5**

The Hardy-Littlewood maximal operator *Mf* is defined by

$$\begin{aligned} M(f)(x)=\sup _{ B\ni x}&\frac{1}{|B|}\int _B|f(y)|dy. \end{aligned}$$

We set $M_\delta (f)=M(|f|^\delta )^{1/\delta },$ where $0<\delta <\infty .$

The sharp maximal operator $M^\sharp f$ is defined by

$$\begin{aligned} M^\sharp (f)(x)=\sup _{ B\ni x}\frac{1}{|B|}\int _B|f(y)-&f_{B}|dy\approx \sup _{ B\ni x}\inf _c\frac{1}{|B|}\int _B|f(y)-c|dy. \end{aligned}$$

We defined the $\delta$- sharp maximal operator $M_\delta ^\sharp (f)=M^\sharp (|f|^\delta )^{1/\delta },$ where $0<\delta <1.$

### **Lemma 2**

(Stein 1993) *Let*$0<q<\infty ,$*and*$\omega \in A_p ( 1\le p<\infty ).$*Then*

$$\begin{aligned} \Vert M_\delta f\Vert _{L^q(\omega )}\lesssim \Vert M_\delta ^\sharp f\Vert _{L^q(\omega )}. \end{aligned}$$

### **Definition 6**

For $0\le \alpha <n,t\ge 1,$ we define the fractional maximal operator $M_{\alpha ,t}f$ by

$$\begin{aligned} M_{\alpha ,t}(f)(x)=\sup _{ B\ni x}\left( \frac{1}{|B|^{1-\alpha t/n}}\int _B|f(y)|^t dy\right) ^{1/t}, \end{aligned}$$

and define the fractional weighted maximal operator $M_{\alpha ,r,\omega }f$ by

$$\begin{aligned} M_{\alpha ,t,\omega }f(x)=\sup _{ B\ni x}\left( \frac{1}{\omega (B)^{1-\alpha t/n}}\int _B|f(y)|^t\omega (y) dy\right) ^{1/t}, \end{aligned}$$

In order to simplify the notation, we set $M_{\alpha }=M_{\alpha ,1},M_{t,\omega }=M_{0,t,\omega }.$

### **Definition 7**

For $0<\alpha <n,$ the fractional integral operator $I_\alpha$ is defined by

$$\begin{aligned} I_\alpha (f)(x)=\int _{{\mathbb {R}}^n}\frac{f(y)}{|x-y|^{n-\alpha }}dy. \end{aligned}$$

### **Lemma 3**

*Let*$I_\alpha$*be fractional integral operator, and letEbe a measurable set in*${\mathbb {R}}^n.$*Then for any*$f\in L^1({\mathbb {R}}^n),$*we have*

$$\begin{aligned} \int _E\left| I_\alpha f(x)\right| dx \lesssim \Vert f\Vert _{L^1}|E|^{\alpha /n}. \end{aligned}$$

### *Proof*

Since

$$\begin{aligned}&\left| \{x\in E: |I_\alpha f(x)|>\lambda \}\right| \\&\quad \le \left| \{x\in R^n: |I_\alpha f(x)|>\lambda \}\right| \\&\quad \lesssim \left( \frac{\Vert f\Vert _{L^1}}{\lambda }\right) ^{n/(n-\alpha )}, \end{aligned}$$

we have

$$\begin{aligned} \int _{E}|I_\alpha f(x)|dx&=\ \int _0^\infty \left| \{x\in E: |I_\alpha f(x)|>\lambda \}\right| d\lambda \\ &\le\ \int _0^\infty \min \left\{ C\Big (\frac{\Vert f\Vert _{L^1}}{\lambda }\right) ^{n/(n-\alpha )},\ |E|\Big \}d \lambda \\ &\le \ \int _0^{C\Vert f\Vert _{L^1}|E|^{\alpha /n-1}}|E|d\lambda +\int _{C\Vert f\Vert _{L^1}|E|^{\alpha /n-1}} ^\infty \left( \frac{\Vert f\Vert _{L^1}}{\lambda }\right) ^{n/(n-\alpha )}d\lambda \\ &\lesssim \ \Vert f\Vert _{L^1}|E|^{\alpha /n}. \end{aligned}$$

$$\square $$

### **Lemma 4**

(See Muckenhoupt 1972) *Let*$0<\alpha <n,1/q=1/p-\alpha /n$*and*$\omega \in A_{p.q}.$*Then*

$$\begin{aligned} \Vert I_\alpha (f)\Vert _{L^q(\omega ^q)}\lesssim \Vert f\Vert _{L^p(\omega ^p)},\ {\text{ and }} \ \Vert M_\alpha (f)\Vert _{L^q(\omega ^q)}\lesssim \Vert f\Vert _{L^p(\omega ^p)}. \end{aligned}$$

Let us recall the weighted Lipschitz function space.

### **Definition 8**

For $1\le p<\infty , 0<\beta <1,$ and $\omega \in A_{\infty }.$ A locally integrable function *b* is said to be in the weighted Lipschitz function space if

4 $$\begin{aligned} \sup _{B}\frac{1}{\omega (B)^{\beta /n}}\left[ \frac{1}{\omega (B)}\int _B|b(x)-b_B|^p \omega (x)^{1-p}dx\right] ^{{1}/{p}}\le C<\infty , \end{aligned}$$

where $b_B=|B|^{-1}\int _Bb(y)dy,$ and the supremum is taken over all balls $B\subseteq {\mathbb {R}}^n.$

The Banach space of such functions modulo constants is denoted by $Lip_{\beta ,p}(\omega ).$ The smallest bound *C* satisfying conditions above is then taken to be the norm of *b* denoted by $\Vert b\Vert _{Lip_{_{\beta ,p}}(\omega )}.$ Obviously, for the case $\omega =1$, the $Lip_{_{\beta ,p}}(\omega )$ space is the classical $Lip_\beta ({\mathbb {R}}^n)$ space. Put $Lip_{\beta }(\omega )= Lip_{\beta ,1}(\omega ).$ Let $\omega \in A_1$. Garcia-Cuerva (1979) proved that the spaces $Lip_{\beta ,p}(\omega )$ coincide, and the norms $\Vert b\Vert _{Lip_{_{\beta ,p}}(\omega )}$ are equivalent with respect to different values of *p* provided that $1\le p<\infty .$ Since we always discuss under the assumption $\omega \in A_1$ in the following, then we denote the norm of $Lip_{\beta ,p}(\omega )$ by $\Vert \cdot \Vert _{Lip_{\beta }(\omega )}$ for $1\le p<\infty .$

## The sharp estimates for $T^b$

To prove our main result, we first prove the following the sharp estimates for $T^b$.

### **Theorem 2**

*Suppose that*$T^b$*is a Toeplitz type operator associated to generalized Calderón–Zygmund operator and fractional integral operator*$I_\alpha$, *and*$b\in Lip_{\beta }(\omega )(0<\beta <1)$. *Let*$1<p<r_0<n/\beta ,\mu =\omega ^{r_0/p}\in A_1,r_\omega>\gamma ',t>\frac{(r_\omega -1)\gamma '}{r_\omega -\gamma '}$*and*$\{jC_j\}\in l^1.$*If*$T^1(f)=0$*for any*$f\in L^{p}(\omega ),$*then there exists a constant*$C>0$*such that*

$$\begin{aligned} M_\delta ^\sharp (T^b(f))(x) &\lesssim \ \Vert b\Vert _{Lip_{\beta }(\omega )}\omega (x)^{1-r_0\alpha \beta /n^2}M_{\beta ,t,\mu }(I_\alpha T_2f)(x)\\&+\quad \Vert b\Vert _{Lip_{\beta }(\omega )}\left( \omega (x)^{1-{\alpha }/{n}}M_{\alpha +\beta ,t,\omega }(T_4(f))(x)+\omega (x)^{1+\beta /n}M_{\alpha +\beta }( T_{4}f)(x)\right) \end{aligned}$$

*holds for any*$0<\delta <1.$

### *Proof*

For any ball $B=B(x_0,r_B)$ which contains *x*. Without loss generality, we may assume $T_1$ is a generalized Calderón–Zygmund operator. We write, by $T^1(f)=0,$

$$\begin{aligned} T^b(f)(y)&=\ T_1M^bI_\alpha T_2(f)(y)+ T_3I_\alpha M^b T_4(f)(y)\\ &=\ {\mathcal {U}}^b(y)+{\mathcal {V}}^b(y)={\mathcal {U}}^{b-b_{2B}}(y)+{\mathcal {V}}^{b-b_{2B}}(y), \end{aligned}$$

where

$$\begin{aligned} {\mathcal {U}}^{b-b_{2B}}(y)&=\ T_1M^{(b-b_{2B})\chi _{2B}}I_\alpha T_2(f)(y)+T_1M^{(b-b_{2B})\chi _{(2B)^c}}I_\alpha T_2(f)(y) \\ &=\ {\mathrm {U}}_1(y)+{\mathrm {U}}_2(y), \end{aligned}$$

and

$$\begin{aligned} {\mathcal {V}}^{b-b_{2B}}(y)&=\ T_3I_\alpha M^{(b-b_{2B})\chi _{2B}}T_4(f)(y)+ T_3I_\alpha M^{(b-b_{2B})\chi _{(2B)^c}} T_4(f)(y) \\ &=\ {\mathrm {V}}_1(y)+{\mathrm {V}}_2(y). \end{aligned}$$

Since $$0<\delta <1,$$ then

$$\begin{aligned}&\left( \frac{1}{|B|}\int _B\Big ||T^b(f)(y)|^\delta -|{\mathrm {U}}_2(x_0)+{\mathrm {V}}_2(x_0)|^\delta \Big |dy\right) ^{1/\delta }\\&\quad \le \left( \frac{1}{|B|}\int _B\Big |T^b(f)(y)-{\mathrm {U}}_2(x_0)-{\mathrm {V}}_2(x_0) \Big |^\delta dy\right) ^{1/\delta }\\&\quad \le \left( \frac{1}{|B|}\int _B |{\mathrm {U}}_1(y)|^\delta dy\right) ^{1/\delta }+\left( \frac{1}{|B|}\int _B |{\mathrm {V}}_1(y)|^\delta dy\right) ^{1/\delta }\\&\qquad +\left( \frac{1}{|B|}\int _B |{\mathrm {U}}_2(y)-{\mathrm {U}}_2(x_0)|^\delta dy\right) ^{1/\delta }+\left( \frac{1}{|B|}\int _B |{\mathrm {V}}_2(y)-{\mathrm {V}}_2(x_0)|^\delta dy\right) ^{1/\delta }\\&\quad =\ M_1+M_2+M_3+M_4. \end{aligned}$$

We are going to estimate each terms, respectively. From Chang et al. (2007), we know that $T_1$ is bounded from $L^1$ to $WL^1 ,$ then by Kolmogorov’s inequality and Hölder’s inequality we get

$$\begin{aligned} M_1&=\left( \frac{1}{|B|}\int _B |{\mathrm {U}}_1(y)|^\delta dy\right) ^{1/\delta }\\ &=\left( \frac{1}{|B|}\int _B |T_1M^{(b-b_{2B})\chi _{2B}}I_\alpha T_2(f)(y)|^\delta dy\right) ^{1/\delta }\\ &\lesssim \ \frac{1}{|B|}\int _{2B}|(b(y)-b_{2B})I_\alpha T_2(f)(y)|dy\\ &\lesssim \ \frac{1}{|B|}\left( \int _{2B}|b(y)-b_{2B}|^{t'}\omega (y)^{1-t'}dy\right) ^{1/t'}\left( \int _{2B}|I_\alpha T_2f(y)|^t\omega (y)dy\right) ^{1/t}\\ &\lesssim \ \Vert b\Vert _{Lip_{\beta }(\omega )}\frac{\omega (B)^{1+\beta /n}}{|B|}\left( \frac{1}{\omega (2B)}\int _{2B}|I_\alpha T_2f(y)|^t\omega (y)dy\right) ^{1/t}. \end{aligned}$$

Since $\mu =\omega ^{r_0/p}\in A_1,$ and $r_0>p,$ then

$$\begin{aligned} \mu (2B)\thickapprox \left( \inf _{x\in 2B}\omega (x)\right) ^{r_0/p-1}\omega (2B). \end{aligned}$$

Thus

5 $$\begin{aligned} \left( \frac{1}{\omega (2B)}\int _{2B}|I_\alpha T_2f(y)|^t\omega (y)dy\right) ^{1/t} \lesssim \left( \frac{1}{\mu (2B)}\int _{2B}|I_\alpha T_2f(y)|^t\mu (y)dy\right) ^{1/t}. \end{aligned}$$

Note that

6 $$\begin{aligned} \omega (B)^{\beta /n}&=\ \left(  \inf _{x\in B}\omega (x)\right) ^{-\alpha \beta r_0/n^2} \left( \int _{B}\omega (x)\left(  \inf _{x\in B}\omega (x)\right) ^{\alpha r_0/n}dx\right) ^{\beta /n}\nonumber \\ &\lesssim \left(  \inf _{x\in B}\omega (x)\right) ^{-\alpha \beta r_0/n^2}\mu (2B)^{\beta /n}. \end{aligned}$$

Since $1/r_0>\beta /n,0<\alpha <n,$ we have $1-\alpha \beta r_0/n^2>0.$ Then by $\omega \in A_1,$ (5) and (6) we get

7 $$\begin{aligned}&\ \frac{\omega (B)^{1+\beta /n}}{|B|}\left( \frac{1}{\omega (2B)}\int _{2B}|I_\alpha T_2f(y)|^t\omega (y)dy\right) ^{1/t}\nonumber \\&\quad \lesssim \left(  \inf _{x\in B}\omega (x)\right) ^{1-\alpha \beta r_0/n^2}M_{\beta ,t,\mu }(I_\alpha T_2f)(x)\nonumber \\&\quad \lesssim \omega (x)^{1-\alpha \beta r_0/n^2}M_{\beta ,t,\mu }(I_\alpha T_2f)(x). \end{aligned}$$

Hence

$$\begin{aligned} M_1\lesssim \Vert b\Vert _{Lip_{\beta }(\omega )}\omega (x)^{1-\alpha \beta r_0/n^2}M_{\beta ,t,\mu }(I_\alpha T_2f)(x). \end{aligned}$$

Since $T_3=\pm I,$ by Lemma 3 and Hölder’s inequality, we have

$$\begin{aligned} M_2&\le \frac{1}{|B|}\int _B|T_3I_\alpha M^{(b-b_{2B})\chi _{2B}}T_4(f)(y)|dy\\ &=\frac{1}{|B|}\int _B|I_\alpha M^{(b-b_{2B})\chi _{2B}}T_4(f)(y)|dy\\ &\lesssim \frac{1}{|B|^{1-\alpha /n}}\int _{{\mathbb {R}}^n}|M^{(b-b_{2B})\chi _{2B}}T_4(f)(y)|dy\\ &\lesssim \frac{1}{|B|^{1-\alpha /n}}\left( \int _{2B}|b(y)-b_{2B}|^{t'}\omega (y)^{1-t'}dy\right) ^{1/t'}\left( \int _{2B}|T_4(f)(y)|^t\omega (y)dy\right) ^{1/t}. \end{aligned}$$

Thus, by (4) and (2) we get

$$\begin{aligned} M_2&\lesssim |b\Vert _{Lip_{\beta }(\omega )}\left( \frac{\omega (2B)}{|B|}\right) ^{1-\alpha /n}\left( \frac{1}{\omega (2B)^{1-(\alpha +\beta ) t/n}}\int _{2B}|T_4(f)(y)|^t\omega (y)dy\right) ^{1/t}\\ &\lesssim |b\Vert _{Lip_{\beta }(\omega )}\omega (x)^{1-\alpha /n}M_{\alpha +\beta ,t,\omega }(T_4f)(x). \end{aligned}$$

By the definition of generalized Calderón–Zygmund operator, we get,

$$\begin{aligned} |{\mathrm {U}}_2(y)-{\mathrm {U}}_2(x_0)|&=\ |T_1M^{(b-b_{2B})\chi _{(2B)^c}}I_\alpha T_2(f)(y)-T_1M^{(b-b_{2B})\chi _{(2B)^c}}I_\alpha T_2(f))(x_0)|\\ &\lesssim \ \int _{(2B)^c}|b(z)-b_{2B}||K(y,z)-K(x_0,z)||I_\alpha T_2(f)(z)|dz. \end{aligned}$$

From Hölder’s inequality we get

$$\begin{aligned} M_3&=\left( \frac{1}{|B|}\int _B |{\mathrm {U}}_2(y)-{\mathrm {U}}_2(x_0)|^\delta dy\right) ^{1/\delta }\\ &\lesssim \ \frac{1}{|B|}\int _B |{\mathrm {U}}_2(y)-{\mathrm {U}}_2(x_0)| dy\\ &\lesssim \ \frac{1}{|B|}\int _B\int _{(2B)^c}|b(z)-b_{2B}||K(y,z)-K(x_0,z)||I_\alpha T_2(f)(z)|dzdy\\ &\lesssim \ \frac{1}{|B|}\sum _{j=1}^\infty \int _B\int _{2^j|y-x_0|\le |z-x_0|<2^{j+1}|y-x_0|}|b(z)-b_{2^{j+1}B}|\\&\quad \times |K(y,z)-K(x_0,z)||I_\alpha T_2(f)(z)|dzdy\\&\quad + \frac{1}{|B|}\sum _{j=1}^\infty |b_{2^{j+1}B}-b_{2B}|\\&\quad \times \int _B\int _{2^j|y-x_0|\le |z-x_0|<2^{j+1}|y-x_0|}|K(y,z)-K(x_0,z)||I_\alpha T_2(f)(z)|dzdy\\ &=\ M_{31}+M_{32}. \end{aligned}$$

For $r_\omega>\gamma '>1,$ we have $t>\frac{(r_\omega -1)\gamma '}{r_\omega -\gamma '}>\gamma ',$ then there exists $1<l<\infty$ such that $1/\gamma +1/l+1/t=1.$ By Hölder’s inequality for $\gamma ,l,$ and *t*,  and (3) of Definition 1 we get

$$\begin{aligned} M_{31}&\lesssim \ \frac{1}{|B|}\sum _{j=1}^\infty \int _B\left( \int _{2^j|y-x_0|\le |z-x_0|<2^{j+1}|y-x_0|}|K(y,z)-K(x_0,z)|^\gamma dz\right) ^{1/\gamma }\\&\quad \times \ \left( \int _{2^j|y-x_0|\le |z-x_0|<2^{j+1}|y-x_0|}|b(z)-b_{2^{j+1}B}|^l\omega (z)^{(1/t'-1)l}dz\right) ^{1/l}\\&\quad \times \left( \int _{2^j|y-x_0|\le |z-x_0|<2^{j+1}|y-x_0|}|I_\alpha T_2(f)(z)|^t\omega (z)dz\right) ^{1/t}dy\\ &\lesssim \ \frac{1}{|B|}\sum _{j=1}^\infty C_j\int _B (2^j|y-x_0|)^{-n/\gamma '}dy\left( \int _{2^{j+1}B}|b(z)-b_{2^{j+1}B}|^l\omega (z)^{(1/t'-1)l}dz\right) ^{1/l}\\&\quad \times \ \left( \int _{2^{j+1}B}|I_\alpha T_2 (f)(z)|^t\omega (z)dz\right) ^{1/t}. \end{aligned}$$

Note that

$$\begin{aligned}\int _B (2^j|y-x_0|)^{-n/\gamma '}dy\lesssim |2^{j}B|^{-1/\gamma '}|B|,\end{aligned}$$

then

$$\begin{aligned} M_{31}&\lesssim \ \sum _{j=1}^\infty C_j|2^{j}B|^{-1/\gamma '}\omega (2^jB)^{1/t}\left( \frac{1}{\omega (2^{j+1}B)}\int _{2^{j+1}B}|I_\alpha T_2 (f)(z)|^t\omega (z)dz\right) ^{1/t}\\&\quad \times \left( \int _{2^{j+1}B}|b(z)-b_{2^{j+1}B}|^l\omega (z)^{(1/t'-1)l}dz\right) ^{1/l}. \end{aligned}$$

Since $t>\frac{(r_\omega -1)\gamma '}{r_\omega -\gamma '},$ we have $r_\omega >\frac{(t-1)\gamma '}{t-\gamma '}.$ By $r_\omega =\sup \{s>1:\omega \in RH_s\},$ there is a *s* such that $s>\frac{(t-1)\gamma '}{t-\gamma '}>\gamma '>1.$ Let $p_0=\frac{s-1}{\frac{(t-1)\gamma '}{t-\gamma '}-1},$ then $1<p_0<\infty .$ By $\frac{1}{\gamma }+\frac{1}{l}+\frac{1}{t}=1$ we have $\frac{l}{t'}=\frac{(t-1)\gamma '}{t-\gamma '}.$ Then $p_0=\frac{s-1}{\frac{l}{t'}-1},$ it is $s=\frac{lp_0}{t'}-\frac{p_0}{p'_0}.$ Applying Hölder’s inequality for $p_0$ and $p'_0$ we get

$$\begin{aligned}&\ \left( \int _{2^{j+1}B}|b(z)-b_{2^{j+1}B}|^l\omega (z)^{({1}/{t'}-1)l}dz\right) ^{{1}/{l}}\\&\quad \lesssim \left( \int _{2^{j+1}B}|b(z)-b_{2^{j+1}B}|^{lp'_0}\omega (z)^{1-lp'_0}dz\right) ^{{1}/{(p'_0l)}}\left( \int _{2^{j+1}B}\omega (z)^sdz\right) ^{{1}/{(p_0l)}}. \end{aligned}$$

Then by (4) and $\omega \in RH_s$

$$\begin{aligned}&\ \left( \int _{2^{j+1}B}|b(z)-b_{2^{j+1}B}|^l\omega (z)^{({1}/{t'}-1)l}dz\right) ^{{1}/{l}}\\&\quad \lesssim \Vert b\Vert _{Lip_{\beta }(\omega )}|2^{j+1}B|^{{(1-s)}/{(p_0l)}}\omega (2^{j+1}B)^{{1}/({p'_0l)}+{s}/({p_0l)}+\beta /n}. \end{aligned}$$

Note that ${(1-s)}/{(p_0l)}-{1}/{\gamma '}=-1$ and ${1}/{t}+{1}/{(p'_0l)}+{s}/{(p_0l)}=1.$ Then, by (6) we get

$$\begin{aligned} M_{31}&\lesssim \ |b\Vert _{Lip_{\beta }(\omega )}\sum _{j=1}^\infty C_j|2^{j+1}B|^{{(1-s)}/{(p_0l)}-{1}/{\gamma '}}\omega (2^{j+1}B)^{{1}/{t}+{1}/{(p'_0l)}+{s}/{(p_0l)}+\beta /n}\\&\quad \times \ \left( \frac{1}{\omega (2^{j+1}B)}\int _{2^{j+1}B}|I_\alpha T_2 (f)(z)|^t\omega (z)dz\right) ^{1/t}\\ &\lesssim \ |b\Vert _{Lip_{\beta }(\omega )}\sum _{j=1}^\infty C_j\frac{\omega (2^{j+1}B)^{1+\beta /n}}{|2^{j+1}B|}\left( \frac{1}{\omega (2^{j+1}B)}\int _{2^{j+1}B}|I_\alpha T_2 (f)(z)|^t\omega (z)dz\right) ^{1/t}\\ &\lesssim \ \Vert b\Vert _{Lip_{\beta }(\omega )}\omega (x)^{1-\alpha \beta r_0/n^2}M_{\beta ,t,\mu }(I_\alpha T_2f)(x)\sum _{j=1}^\infty C_j\\ &\lesssim \ \Vert b\Vert _{Lip_{\beta }(\omega )}\omega (x)^{1-\alpha \beta r_0/n^2}M_{\beta ,t,\mu }(I_\alpha T_2f)(x). \end{aligned}$$

Let’s estimate $M_{32}.$ By (6), when $k\le j$ we have

8 $$\begin{aligned} \omega (2^{k+1}B)^{\beta /n}&\lesssim \ \inf _{x\in 2^{k+1}B}\omega (x)^{-\alpha \beta r_0/n^2}\mu (2^{k+1}B)^{\beta /n}\nonumber \\ &\lesssim \ \inf _{x\in 2^{k+1}B}\omega (x)^{-\alpha \beta r_0/n^2}\mu (2^{j+1}B)^{\beta /n}. \end{aligned}$$

Then

9 $$\begin{aligned} |b_{2^{j+1}B}-b_{2B}| &\le \ \sum _{k=1}^j\frac{1}{|2^{k}B|}\int _{2^{k+1}B}|b(z)-b_{2^{k+1}B}|dz\nonumber \\ &\lesssim \ \Vert b\Vert _{Lip_{\beta }(\omega )}\sum _{k=1}^j\frac{\omega (2^{k+1}B)^{1+{\beta }/{n}}}{|2^{k+1}B|}\nonumber \\ &\lesssim \ \Vert b\Vert _{Lip_{\beta }(\omega )}\sum _{k=1}^j\inf _{x\in 2^{k+1 }B}\omega (x)\omega (2^{k+1}B)^{\beta /n}\nonumber \\ &\lesssim \ \Vert b\Vert _{Lip_{\beta }(\omega )}\sum _{k=1}^j\inf _{x\in 2^{k+1 }B}\left( \omega (x)\right) ^{1-{\alpha \beta r_0}/{n^2}}\mu (2^{j}B)^{\beta /n}\nonumber \\ &\lesssim \ j\Vert b\Vert _{Lip_{\beta }(\omega )}\omega (x)^{1-{\alpha \beta r_0}/{n^2}}\mu (2^{j}B)^{\beta /n}. \end{aligned}$$

Hence, by Hölder’s inequality, (8) and (9), we can get

$$\begin{aligned} M_{32}&\lesssim \ \frac{1}{|B|}\sum _{j=1}^\infty \int _B\left( \int _{2^j|y-x_0|\le |z-x_0|<2^{j+1}|y-x_0|}|K(y,z)-K(x_0,z)|^\gamma dz\right) ^{1/\gamma }\\&\quad \times \ |b_{2^{j+1}B}-b_{2B}||2^{j+1}B|^{1/l} \left( \int _{2^{j+1}B}|I_\alpha T_2(f)(z)|^tdz\right) ^{1/t}dy\\ &\lesssim \ \Vert b\Vert _{Lip_{\beta }(\omega )}\omega (x)^{1-{\alpha \beta r_0}/{n^2}}\sum _{j=1}^\infty jC_j\int _B (2^j|y-x_0|)^{-n/\gamma '}dy\\&\quad \times \ \mu (2^{j}B)^{\beta /n}\left( \frac{1}{\omega (2^{j+1}B)}\int _{2^{j+1}B}|I_\alpha T_2 (f)(z)|^t\omega (z)dz\right) ^{1/t}|2^{j+1}B|^{1/l+1/t}\\ &\lesssim \ \Vert b\Vert _{Lip_{\beta }(\omega )}\omega (x)^{1-{\alpha \beta r_0}/{n^2}}M_{\beta ,t,\mu }(I_\alpha T_2f)(x)\sum _{j=1}^\infty jC_j|2^{j+1}B|^{1/l+1/t-1/\gamma '}\\ &\lesssim \ \Vert b\Vert _{Lip_{\beta }(\omega )}\omega (x)^{1-{\alpha \beta r_0}/{n^2}}M_{\beta ,t,\mu }(I_\alpha T_2f)(x). \end{aligned}$$

Then

$$\begin{aligned} M_3\lesssim \Vert b\Vert _{Lip_{\beta }(\omega )}\omega (x)^{1-{\alpha \beta r_0}/{n^2}}M_{\beta ,t,\mu }(I_\alpha T_2f)(x). \end{aligned}$$

Finally, we estimate $M_4.$ For any $y\in B,$ and $z\in (2B)^c,$ we have $|y-z|\sim |x_0-z|.$ Then,

$$\begin{aligned} M_4&=\ \left( \frac{1}{|B|}\int _B |{\mathrm {V}}_2(y)-{\mathrm {V}}_2(x_0)|^\delta dy\right) ^{1/\delta }\\ &\le \ \frac{1}{|B|}\int _B |{\mathrm {V}}_2(y)-{\mathrm {V}}_2(x_0)|dy \\ &\le \ \frac{1}{|B|}\int _{B} |T_{3}I_\alpha M^{(b-b_{2B})\chi _{(2B)^c}} T_{4}(f)(y)-T_{3}I_\alpha M^{(b-b_{2B})\chi _{(2B)^c}} T_{4}(f)(x_0)|dy\\ &\lesssim \ \frac{1}{|B|}\int _{B}\int _{(2B)^c}|b(z)-b_{2B}|\left| \frac{1}{|y-z|^{n-\alpha }}-\frac{1}{|x_0-z|^{n-\alpha }}\right| | T_{4}(f)(z)|dzdy\\ &\lesssim \ \frac{1}{|B|}\int _{B}\int _{(2B)^c}|b(z)-b_{2B}|\frac{|x_0-y|}{|x_0-z|^{n-\alpha +1}} | T_{4}(f)(z)|dzdy\\ &\lesssim \ \sum _{j=1}^\infty \frac{r}{(2^jr)^{n-\alpha +1}}\int _{2^{j+1}B}|b(z)-b_{2B}|| T_{4}(f)(z)|dz\\ &\lesssim \ \sum _{j=1}^\infty 2^{-j}|b_{2^{j+1}B}-b_{2B}|\frac{1}{|2^{j+1}B|^{1-\alpha /n}}\int _{2^{j+1}B}| T_{4}(f)(z)|dz\\&\quad + \sum _{j=1}^\infty 2^{-j}\frac{1}{|2^{j+1}B|^{1-\alpha /n}}\int _{2^{j+1}B}|b(z)-b_{2^{j+1}B}|| T_{4}(f)(z)|dz\\ &=\ M_{41}+N_{42}. \end{aligned}$$

Since

$$\begin{aligned} |b_{2^{j+1}B}-b_{2B}| &\lesssim \ \Vert b\Vert _{Lip_{\beta }(\omega )}\sum _{k=1}^j\inf _{x\in 2^{k+1 }B}\omega (x)\omega (2^{k+1}B)^{{\beta }/{n}}\\ &\lesssim \ j\Vert b\Vert _{Lip_{\beta }(\omega )}\omega (x)\left(  \inf _{x\in 2^{j+1}B}\omega (x)\right) ^{\beta /n}|2^{j+1}B|^{\beta /n}\\ &\lesssim \ j\Vert b\Vert _{Lip_{\beta }(\omega )}\omega (x)^{1+\beta /n}|2^{j+1}B|^{\beta /n}, \end{aligned}$$

then

$$\begin{aligned} M_{41}&\lesssim \ \sum _{j=1}^\infty 2^{-j}|b_{2^{j+1}B}-b_{2B}|\frac{1}{|2^{j+1}B|^{1-\alpha /n}}\int _{2^{j+1}B}| T_{4}(f)(z)|dz\\ &\lesssim \ \Vert b\Vert _{Lip_{\beta }(\omega )}\omega (x)^{1+\beta /n}M_{\alpha +\beta }( T_{4}f)(x)\sum _{j=1}^\infty j2^{-j}\\ &\lesssim \ \Vert b\Vert _{Lip_{\beta }(\omega )}\omega (x)^{1+\beta /n}M_{\alpha +\beta }( T_{4}f)(x). \end{aligned}$$

By Hölder’s inequality,

$$\begin{aligned} M_{42}&\lesssim \sum _{j=1}^\infty 2^{-j}\frac{1}{|2^{j+1}B|^{1-\alpha /n}}\int _{2^{j+1}B}|b(z)-b_{2^{j+1}B}|| T_{4}(f)(z)|dz\\ &\lesssim \ \sum _{j=1}^\infty 2^{-j}\frac{1}{|2^{j+1}B|^{1-{\alpha }/{n}}}\left( \int _{2^{j+1}B} |b(z)-b_{2^{j+1}B}|^{t'}\omega (z)^{1-t'}dz\right) ^{{1}/{{t'}}}\\&\quad \times \left( \int _{2^{j+1}B}| T_4(f)(z)|^{t}\omega (z)dz\right) ^{{1}/{{t}}}\\ &\lesssim \ \Vert b\Vert _{Lip_{\beta }(\omega )}\sum _{j=1}^\infty 2^{-j}\left( \frac{\omega (2^{j+1}B)}{|2^{j+1}B|}\right) ^{1-{\alpha }/{n}}\\&\quad \times \left( \frac{1}{\omega (2^{j+1}B)^{1-{(\alpha +\beta )t}/{n}}}\int _{2^{j+1}B}| T_4(f)(z)|^{t}\omega (z)dz\right) ^{{1}/{{t}}}\\ &\lesssim \ \Vert b\Vert _{Lip_{\beta }(\omega )}\omega (x)^{1-{\alpha }/{n}}M_{\alpha +\beta ,t,\omega }(T_4(f))(x). \end{aligned}$$

Then

$$\begin{aligned} M_4\lesssim \Vert b\Vert _{Lip_{\beta }(\omega )}\left( \omega (x)^{1-{\alpha }/{n}}M_{\alpha +\beta ,t,\omega }(T_4f)(x)+\omega (x)^{1+\beta /n}M_{\alpha +\beta }( T_{4}f)(x)\right) . \end{aligned}$$

Combining the estimates for $M_1,M_2,M_3$ and $M_4,$ the proof of Theorem 2 is completed. $\square$

## Proof of Theorem 1

### *Proof*

It follows from $r_\omega >\frac{(q-1)\gamma '}{q-\gamma '}$ that $q>\frac{(r_\omega -1)\gamma '}{r_\omega -\gamma '},$ then there exists *t* such that $q>t>\frac{(r_\omega -1)\gamma '}{r_\omega -\gamma '}.$ Let $1/q=1/r_0-\beta /n,$ and $1/r_0=1/p-\alpha /n.$ Then

$$\begin{aligned} (1-{\alpha \beta r_0}/{n^2})q+1-(1-\alpha /n)q=r_0/p, \end{aligned}$$

and

$$\begin{aligned} (1+\beta /n)q+1-(1-\alpha /n)q=q/p. \end{aligned}$$

Since $\omega ^{q/p}\in A_1,$ then $\mu =\omega ^{r_0/p}\in A_1,$ and $\omega \in A_1.$ By (1) we have $\omega ^{1/p}\in A_{p,r_0}$ and $\omega ^{1/p}\in A_{p,q}.$ Thus, by Lemma 2, Theorem 2, Lemma 4 and the boundedness of $T_2, T_4$ on $L^p(\omega ),$ we have

$$\begin{aligned}&\ \Vert T_b(f)\Vert _{L^q(\omega ^{1-(1-\alpha /n)q})}\\ &\lesssim \ \Vert M_\delta ^\sharp T_b(f)\Vert _{L^q(\omega ^{1-(1-\alpha /n)q})}\\ &\lesssim \ \Vert b\Vert _{Lip_{\beta }(\omega )}\Big ( \left\| \omega (\cdot )^{1-{\alpha \beta r_0}/{n^2}} M_{\beta ,t,\mu }(I_\alpha T_2f)\right\| _{L^q(\omega ^{1-(1-\alpha /n)q})}\\&\quad +\left\| \omega (\cdot)^{1-\alpha /n}M_{\alpha +\beta ,t,\omega }(T^{k,4}f)\right\| _{L^q(\omega ^{1-(1-\alpha /n)q})}\\&\quad +\left\| \omega (\cdot )^{1+{\beta }/{n}}M_{\alpha +\beta }(T_4(f))\right\| _{L^q(\omega ^{1-(1-\alpha /n)q})}\Big )\\ &\lesssim \ \Vert b\Vert _{Lip_{\beta }(\omega )}\Big ( \left\| M_{\beta ,t,\mu }(I_\alpha T_2f)\right\| _{L^q(\mu )}\\&\quad  + \Vert M_{\alpha +\beta ,t,\omega }(T_4f)\Vert _{L^{q}(\omega )}+\Vert M_{\alpha +\beta }(T_4(f))\Vert _{L^q(\omega ^{q/p})}\Big )\\ &\lesssim \ \Vert b\Vert _{Lip_{\beta }(\omega )}\left( \Vert I_\alpha T_2f\Vert _{L^{r_0}(\mu )}+\Vert T_4(f)\Vert _{L^p(\omega )}\right) \\ &\lesssim \ \Vert b\Vert _{Lip_{\beta }(\omega )}\Vert f\Vert _{L^p(\omega )}. \end{aligned}$$

This finishes the proof of Theorem 1. $\square$

## Conclusion

In the present paper, we have established the sharp maximal function estimates for a class of Toeplitz type operator and we have obtained the weighted norm inequalities related to the operators on weighted Lebesgue space. We believe that these results are object of interest for a lots of scientists that study regularity of solutions of partial differential equations.
